# Meta-regression of genome-wide association studies to estimate age-varying genetic effects

**DOI:** 10.1007/s10654-023-01086-1

**Published:** 2024-01-06

**Authors:** Panagiota Pagoni, Julian P. T. Higgins, Deborah A. Lawlor, Evie Stergiakouli, Nicole M. Warrington, Tim T. Morris, Kate Tilling

**Affiliations:** 1 MRC Integrative Epidemiology Unit at the University of Bristol, Oakfield House, Oakfield Grove, Bristol, BS8 2BN UK; 2 Population Health Sciences, Bristol Medical School, University of Bristol, Bristol, UK; 3 Institute for Molecular Bioscience, University of Queensland, Brisbane, QLD Australia; 4 Frazer Institute, University of Queensland, Brisbane, QLD Australia; 5 K.G. Jebsen Center for Genetic Epidemiology, Department of Public Health and Nursing, NTNU, Norwegian University of Science and Technology, Trondheim, Norway; 6 Centre for Longitudinal Studies, Social Research Institute, University College London, London, UK

**Keywords:** Genome-wide association studies, Meta-analysis, Meta-regression, Age-varying genetic effects

## Abstract

Fixed-effect meta-analysis has been used to summarize genetic effects on a phenotype across multiple Genome-Wide Association Studies (GWAS) assuming a common underlying genetic effect. Genetic effects may vary with age (or other characteristics), and not allowing for this in a GWAS might lead to bias. Meta-regression models between study heterogeneity and allows effect modification of the genetic effects to be explored. The aim of this study was to explore the use of meta-analysis and meta-regression for estimating age-varying genetic effects on phenotypes. With simulations we compared the performance of meta-regression to fixed-effect and random -effects meta-analyses in estimating (i) main genetic effects and (ii) age-varying genetic effects (SNP by age interactions) from multiple GWAS studies under a range of scenarios. We applied meta-regression on publicly available summary data to estimate the main and age-varying genetic effects of the *FTO* SNP rs9939609 on Body Mass Index (BMI). Fixed-effect and random-effects meta-analyses accurately estimated genetic effects when these did not change with age. Meta-regression accurately estimated both main genetic effects and age-varying genetic effects. When the number of studies or the age-diversity between studies was low, meta-regression had limited power. In the applied example, each additional minor allele (A) of rs9939609 was inversely associated with BMI at ages 0 to 3, and positively associated at ages 5.5 to 13. Our findings challenge the assumption that genetic effects are consistent across all ages and provide a method for exploring this. GWAS consortia should be encouraged to use meta-regression to explore age-varying genetic effects.

**Supplementary Information:**

The online version contains supplementary material available at 10.1007/s10654-023-01086-1.

## Introduction

Genome–wide association studies (GWAS) test associations of millions of single nucleotide polymorphisms (SNPs) across the genome with a phenotype. As SNP effects are generally small, large sample sizes are required for adequate statistical power. This is commonly achieved through fixed-effect meta-analysis of summary genetic effects across several GWAS, which increases sample size and statistical power without sharing individual participant data.

Fixed-effect meta-analysis, which assumes a common true underlying genetic effect for all studies [1], has been favored over random-effects meta-analysis due to its increased statistical power [2]. Fixed-effect meta-analysis ignores heterogeneity of genetic effects between studies, and it has been suggested that this could introduce high rates of false positive and/or false negative findings under certain conditions [2, 3]. One such condition is where genetic effects vary with age, as they have been demonstrated to do for some phenotypes [4–8]. As meta-analysis estimates an average genetic effect over all studies, meta-analysing GWAS studies of age-diverse samples, without considering potential heterogeneity of genetic effects due to age, could fail to identify clinically important changes of genetic risk with age. Moreover, ignoring age-varying genetic effects in GWAS may lead to spurious results in other methods that use GWAS summary data as input to estimate: genetic correlation between traits (LD score regression) [9], genetic predisposition to a trait (Polygenic Risk Scores) [10] and the causal effect of an exposure on an outcome (Two-sample Mendelian randomization) [11].

An approach recommended in meta-analysis of Randomized Controlled Trials (RCTs) to estimate treatment-covariate interactions (e.g., treatment-age interactions) is a two-stage approach, where the interactions are estimated within each study, and these interactions are then meta-analysed [12]. This approach would have limited application in GWAS as most studies do not perform or report an interaction analysis (e.g., SNP-age interaction effects).

An alternative method that could be used to overcome this issue is meta-regression. In contrast to meta-analysis that uses summary GWAS data to estimate an average genetic effect over all studies, meta-regression models observed heterogeneity and allows investigation of the impact of moderator variables on estimated genetic effect sizes [13, 14]. For example, considering age as the moderator variable meta-regression could be used to estimate not only the average genetic effect at the weighted mean age over all studies or at any age of interest, but also the age-varying genetic effect (i.e., SNP by age interaction). A search of the literature and GWAS data bases in July 2022, suggests that meta-regression has been applied in only two GWASs to explore age related differences between included studies. In both cases, genetic variants with age-varying genetic effects were identified [8, 15]. We have not identified published research exploring the conditions under which meta-regression outperforms meta-analysis when age-varying genetic effects exist.

The aim of this study was to explore the use of meta-analysis and meta-regression to examine age-varying genetic effects on phenotypes, using summary GWAS data. We compared the performance of meta-regression and fixed-effect and random-effects meta-analysis in estimating (i) main genetic effects (i.e., the SNP effect at the youngest age available across all studies, age 10) and (ii) age- varying genetic effects (SNP by age interactions) using multiple simulated cross-sectional GWAS studies. We simulated phenotype-genotype associations under a range of data generating processes, varying the number of studies and their sample sizes, the overlap in the age range of study participants between studies (i.e., age-diversity), and the sampling variability within and between studies. We also present an empirical example in which we applied meta-analysis and meta-regression to estimate the age-varying genetic associations between the rs9939609 SNP at the *FTO* locus and body mass index (BMI) across early life-course, using publicly available summary data, and compare these to estimates from previous individual-participant analyses.

## Methods

### Data generating mechanisms for simulations

Participant age (${age}_{ij}\text{ for participant }i\text{ in study }j$), drawn from a uniform distribution, was set between 10 and 59 years. A single SNP with a large effect size, ${SNP}_{ij}$, was simulated with a minor allele frequency (MAF) of 0.2 and the number of risk alleles (0,1,2) was drawn from a binomial distribution. We generated the outcome phenotype (${Y}_{ij}$) to be dependent on: *Scenario 1*. age and genotype; *Scenario 2*. age and genotype, with an interaction between age and genotype (linear interaction term); *Scenario 3.* age, genotype and a quadratic term of age; *Scenario 4.* genotype, age and a quadratic term of age, with an interaction between age and genotype (linear interaction term); *Scenario 5*. genotype, age and a quadratic term of age, where genotype interacts with age and quadratic age (non-linear interaction term). Equations for the phenotype generating scenarios and the parameter values are presented in Table 1. We assumed that the effect of age on phenotype (${\beta }_{age}$) was identical for each study but that the effect of genotype varied randomly across studies (${\beta }_{SNP}+{u}_{j}$), corresponding to a random-effects meta-analysis model for the genotype–phenotype association. As a “base case” scenario, we used 1 SD within and between study variability (${\varepsilon }_{ij} \sim N\left(\mathrm{0,1}\right)$ and ${u}_{j}\sim N\left(\mathrm{0,1}\right)$) in the data generating mechanisms, with 40 cross-sectional studies each with sample size ${N}_{j}=1000$.

**Table 1:** Equations underlying the phenotype generating mechanism for each simulated scenario and parameter values for ‘Base case scenario’

Simulated scenarios	
Scenario 1	${Y}_{ij}={\beta }_{0}+{\beta }_{age}\times {age}_{ij}+{{(\beta }_{SNP}+{u}_{j})\times SNP}_{ij}+{\varepsilon }_{ij}$
Scenario 2	${Y}_{ij}={\beta }_{0}+{\beta }_{age}\times {age}_{ij}+{{(\beta }_{SNP}+{u}_{j})\times SNP}_{ij}+{{{\beta }_{SNP\times age}\times {age}_{ij}\times SNP}_{ij}+ \varepsilon }_{ij}$
Scenario 3	${Y}_{ij}={\beta }_{0}+{\beta }_{age}\times {age}_{ij}+{\beta }_{{age}^{2}}\times {age}_{ij}^{2}+{{(\beta }_{SNP}+{u}_{j})\times SNP}_{ij}+{\varepsilon }_{ij}$
Scenario 4	${Y}_{ij}={\beta }_{0}+{\beta }_{age}\times {age}_{ij}+{\beta }_{{age}^{2}}\times {age}_{ij}^{2}+{{(\beta }_{SNP}+{u}_{j})\times SNP}_{ij}+{\beta }_{SNP\times age}{{\times {age}_{ij}\times SNP}_{ij}+ \varepsilon }_{ij}$
Scenario 5	${Y}_{ij}={\beta }_{0}+{\beta }_{age}\times {age}_{ij}+{\beta }_{{age}^{2}}\times {age}_{ij}^{2}+{{(\beta }_{SNP}+{u}_{j})\times SNP}_{ij}+{{{\beta }_{SNP\times age}\times {age}_{ij}\times SNP}_{ij}+{\beta }_{{SNP\times age}^{2}}\times {age}_{ij}^{2}{\times SNP}_{ij}+ \varepsilon }_{ij}$

Parameter	Value	Interpretation
${\beta }_{0}$	25	Baseline mean value of phenotype when ${age}_{ij}=0$ and ${SNP}_{ij}=0$
${\beta }_{age}$	0.010	Effect of age on phenotype
${age}_{ij}$	~ *U (min age, max age)*	Age of participant $i$ in study $j$
${\beta }_{SNP}$	1.5	Effect of genetic variant on phenotype (main genetic effect)
${SNP}_{ij}$	0,1,2	Number of alleles of a genetic variant for participant $i$ in study $j$
${\beta }_{SNP\times age}$	0.020	Age varying-genetic effect on phenotype (linear interaction term)
${\beta }_{{SNP\times age}^{2}}$	0.001	Non-linear age-varying genetic effect on phenotype (non-linear interaction term)
${\beta }_{{age}^{2}}$	0.001	Non-linear effect of age on phenotype
${\varepsilon }_{ij}$	$\sim N(\mathrm{0,1})$	Within study sampling error
${u}_{j}$	$\sim N(\mathrm{0,1})$	Between study sampling error

jth study j = (1, 2, …,40), ith participant i = (1, 2, …, 1000)

### Estimating study-specific genotype–phenotype associations

Within each cross-sectional study, we used linear regression to estimate the genotype–phenotype association. As is usual in GWAS studies, models were adjusted only for age, and no further adjustments were made to account for non-linearity or SNP-age interactions. Equation (1) describes the regression models:

1 $${Y}_{ij}={\beta }_{0j}+{\beta }_{1j}\times {SNP}_{ij}+{\beta }_{2j}\times {age}_{ij}+{\varepsilon }_{ij}$$

We collected the estimated genotype–phenotype effect estimate (${\widehat{\beta }}_{1j}$) and its standard error ($\widehat{SE({\beta }_{1j})}$) from each study, in addition to the mean age (${\overline{age} }_{\mathrm{j}}$) and standard deviation of age ($SD({age}_{j})$) for each study.

## Description of compared methods

### Meta-analysis

Fixed-effect meta-analysis assumes that all studies draw a (random) sample from the same underlying population and hence share a common true effect size for each SNP. The pooled meta-analysis estimates the population average genetic effect at the weighted mean age over all studies [16]. The observed effect for a given SNP in each study is:

2 $${\beta }_{1j}={\beta }_{SNP}+ {\eta }_{j}, {\eta }_{j} \sim N\left(0,{s}_{j}^{2}\right)$$

where, ${\beta }_{1j}$ the genotype–phenotype effect in the jth study, ${\beta }_{SNP}$ is the common genetic effect at the weighted mean age over all studies, and ${\eta }_{j}$ is random error describing the sampling variability within each study (with variance ${s}_{j}^{2}$ in study *j*, i.e., the variance of ${\beta }_{1j}$).

Random-effects meta-analysis allows the true genetic effect size to differ across studies. The observed effect for a given SNP in each study is:

3 $$\begin{array}{*{20}l} {\beta_{1j} = \beta_{SNP} + \xi_{j} + \eta_{j} ,} \hfill & {\eta_{j} \sim N\left( {0,s_{j}^{2} } \right)} \hfill \\ {} \hfill & { \xi_{j} \sim N\left( {0,\tau^{2} } \right)} \hfill \\ \end{array}$$

where ${\beta }_{SNP}$ is the mean genetic effect at the weighted mean age, ${\xi }_{j}$ represents heterogeneity, i.e. the study-specific deviation from the mean genetic effect (with variance ${\tau }^{2}$ across studies, i.e. the between study variability), and ${\eta }_{j}$ is random error describing the sampling variability within each study (with variance ${s}_{j}^{2}$ in study *j*, i.e. the variance of ${\beta }_{1j}$). Further information about the estimation of combined genetic effects in fixed-effect and random-effects meta-analysis can be found in Online Resource 2. To estimate the between-study variance ${\tau }^{2}$, we used restricted maximum likelihood (REML) method [17].

### Meta-regression

Random-effects meta-regression extends the random-effects meta-analysis model as follows:

4 $$\begin{array}{*{20}l} {\beta_{1j} = \beta_{SNP} + \beta_{SNP \times age} \overline{age}_{{\text{j}}} + \xi_{j} + \eta_{j} , } \hfill & { \eta_{j} \sim N\left( {0,s_{j}^{2} } \right) } \hfill \\ {} \hfill & { \xi_{j} \sim N\left( {0,\tau_{res}^{2} } \right) } \hfill \\ \end{array}$$

and could also be further extended to include non-linear terms such as:

5 $$\begin{array}{*{20}l} {\beta_{1j} = \beta_{SNP} + \beta_{SNP \times age} \overline{age}_{{\text{j}}} + \beta_{{SNP \times age^{2} }} \overline{{age_{j}^{2} }} + \xi_{j} + \eta_{j} ,} \hfill & {\eta_{j} \sim N\left( {0,s_{j}^{2} } \right)} \hfill \\ {} \hfill & {\xi_{j} \sim N\left( {0,\tau_{res}^{2} } \right)} \hfill \\ \end{array}$$

where, ${\beta }_{SNP\times age}$ is the difference in the mean effect of a given SNP for each one year increase in age, ${\beta }_{SNP\times {age}^{2}}$ is the difference in the mean effect of a given SNP for each one year difference in the square of age and ${\tau }_{res}^{2}$ is the residual heterogeneity after accounting for the age effect(s). Meta-regression estimates these two parameters (${\widehat{\beta }}_{SNP\times age}$, ${\widehat{\beta }}_{SNP\times {age}^{2}}$) and an intercept term (${\widehat{\beta }}_{SNP}$) representing the effect of genotype on phenotype for age = 0. In Eq. (5), ${\overline{age} }_{\mathrm{j}}$ is the mean age, $\overline{{age }_{j}^{2}}$ is the mean of quadratic age of each study. Mean of quadratic age is often not available in GWAS, but it can be derived from within-study standard deviation of age, which is often available in GWAS, using the formula ($\overline{{age }_{j}^{2}}\sim {SD\left({age}_{j}\right)}^{2}+ {({\overline{age} }_{\mathrm{j}})}^{2}$). Further information about the derivation of the meta-regression models including quadratic or cubic terms of age can be found in Online Resource 2. To estimate the between-study variance ${\tau }_{res}^{2}$ we used REML [17].

As the effect of genotype on phenotype when age is zero may not be of interest for many phenotypes, meta-regression models can be modified to estimate the effect of genotype on phenotype at a given age of interest (e.g., an age that is within the age range of all studies). Similarly to regression models, including age centred at the age of interest in the meta-regression models will change the estimate of the intercept term (${\widehat{\beta }}_{SNP}$), which will be the effect of genotype on phenotype at the age of interest, while interaction terms will remain unchanged. For example, in our simulations we were interested in estimating the effect of genotype on phenotype at age 10 (the lowest age across studies) and therefore, models (4) and (5) were modified as follows:

6 $$\begin{gathered} \begin{array}{*{20}l} {\beta_{1j} = \beta_{SNP} + \beta_{SNP \times age} \times \left( {\overline{age}_{{\text{j}}} - 10} \right) + \xi_{j} + \eta_{j} ,} \hfill & {\eta_{j} \sim N\left( {0,s_{j}^{2} } \right)} \hfill \\ {} \hfill & {\xi_{j} \sim N\left( {0,\tau_{res}^{2} } \right) } \hfill \\ \end{array} \hfill \\ \hfill \\ \end{gathered}$$

and

7 $$\begin{array}{*{20}l} {\beta_{1j} = \beta_{SNP} + \beta_{SNP \times age} \times (\overline{age}_{{\text{j}}} - 10) + \beta_{{SNP \times age^{2} }} \times \overline{{\left( {age_{j} - 10} \right)^{2} }} + \xi_{j} + \eta_{j} ,} \hfill & {\eta_{j} \sim N\left( {0,s_{j}^{2} } \right) } \hfill \\ {} \hfill & {\xi_{j} \sim N\left( {0,\tau_{res}^{2} } \right)} \hfill \\ \end{array}$$

where, (${\widehat{\beta }}_{SNP})$ is now the effect of genotype on phenotype at age = 10 (referred to as main genetic effect), ${\beta }_{SNP\times age}$ is the difference in the mean effect of a given SNP for each one year increase in age and ${\beta }_{SNP\times {age}^{2}}$ is the difference in the mean effect of a given SNP for each one year difference in the square of age.

It is important to note that centering age at the weighted mean of all studies in meta-regression is expected to yield similar results to meta-analysis only in cases where the phenotype-genotype association does not change, or changes linearly, with age. In that case the (weighted) mean genetic effect across all studies (meta-analysis) will be equivalent to the genetic effect at the (weighted) mean age (centred meta-regression). Additionally, it is expected that standard errors for the main genetic effect will be the lowest when centering age at the mean of all studies in meta-regression, and equivalent to standard errors as estimated in random-effects meta-analysis. However, in our simulations we chose to centre age at the lowest age of all studies rather than at the mean age across all studies, as centring at mean age would have been data-driven and not an age of interest for many phenotypes.

### Implementation

**Fig. 1:**
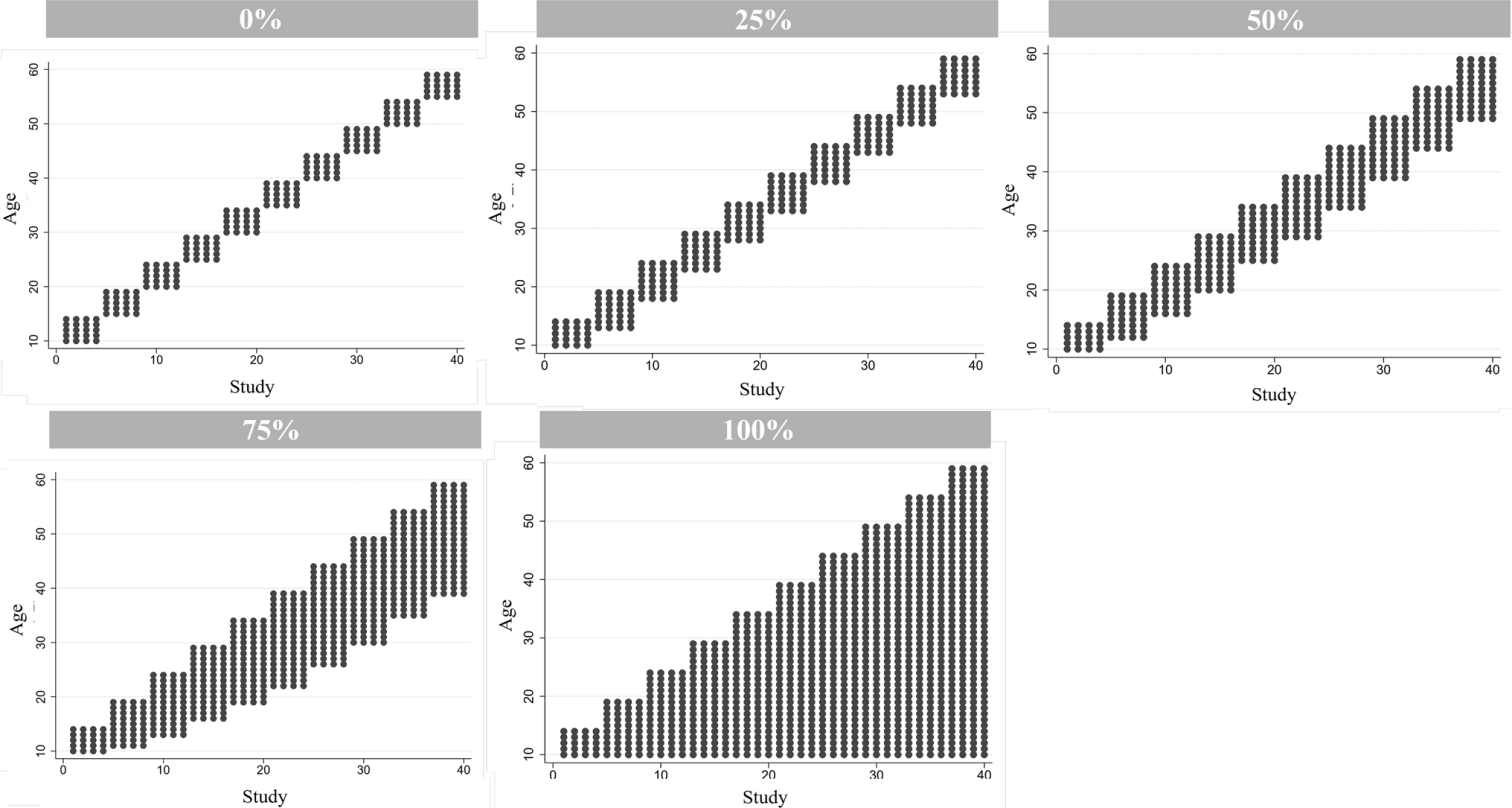
Scatter plot of age of each participant within each study to show age overlap

For each scenario, we ran 1000 iterations. We varied (i) study sample sizes from 1000 to 10,000, (ii) the number of studies from 10 to 80, (iii) the within and between study variability from 1 (${\varepsilon }_{ij}\sim N\left(\mathrm{0,1}\right)$ and ${u}_{j}\sim N(\mathrm{0,1})$) to 3 (${\varepsilon }_{ij}\sim N$(0,${3}^{2}$) and ${u}_{j}\sim N$(0,${3}^{2}$)), and (iv) the overlap of age distributions across studies (i.e., a reflection of the spread of study age means and age-diversity) from no overlap (0%) (i.e., high age-diversity and spread of study age means) to complete overlap (100%) (i.e., minimal age-diversity and spread of study age means) in 25% increments. Figure 1 depicts the overlap of age distributions between studies. We also varied, (v) the age ranges of studies to allow for standard deviations of age to differ between studies. Further information about the age ranges of each study can be found in Online Resource 1 (Table S1 and S27). Lastly, (vi) we run simulations where the simulated value for the main genetic effect is the effect of genotype on phenotype at the weighted mean age across all studies. In this simulated scenario, meta-regression models included age centred at the weighted mean age across all studies, instead of centring age at age 10 as in the previous simulations.

### Estimands and performance measures

The estimands of interest were the main genetic effect (${\upbeta }_{\mathrm{SNP}}$) (the effect of the SNP if the population had mean age = 10), the linear age-varying genetic effect (${\upbeta }_{\mathrm{SNP}\times \mathrm{Age}}$), the non-linear age-varying effect (${\upbeta }_{\mathrm{SNP}\times {Age}^{2}}$), and the standard errors (SE) of these parameters across simulations.

We present five performance measures: the mean estimate, the bias (the deviation of the estimated parameter from the simulated value), the coverage of the 95% confidence interval (CI) (the proportion of simulated datasets for which the 95% confidence interval included the simulated value), the empirical standard error (Emp SE), and the mean standard error (Mean SE).

### Estimating the age-varying genetic association between the rs9939609 SNP at the FTO locus and body mass index (BMI)

We used a real data example to illustrate the application of meta-analysis and meta-regression in estimating age-varying genetic effects using summary level association statistics. An age-varying association between the rs9939609 SNP at the *FTO* locus and body mass index (BMI) has been previously demonstrated [4, 5, 18, 19]. We extracted summary level data for the association between rs9939609 and BMI from a study investigating the effect of this genetic variant on BMI from infancy to late childhood [5]. The effect of rs9939609 on BMI was estimated in 8 cohorts (N = 569 to 7482) at up to 10 ages within each cohort from 0 to 13 years. Detailed information about effect sizes within each cohort at each time point can be found in Online Resource 1 (Table S2).

We estimated the association between rs9939609 SNP and BMI using fixed-effect meta-analysis and meta-regression adjusting for a cubic term of age, which can be written as follows:

8 $$\begin{gathered} \begin{array}{*{20}l} {{\upbeta }_{j} = \beta_{SNP} + \beta_{SNP \times age} \overline{age}_{{\text{j}}} + \beta_{{SNP \times age^{2} }} \overline{{age_{j}^{2} }} + \beta_{{SNP \times age^{3} }} \overline{{age_{j}^{3} }} + \xi_{j} + \eta_{j} , } \hfill & { \eta_{j} \sim N\left( {0,s_{j}^{2} } \right) } \hfill \\ {} \hfill & { \xi_{j} \sim N\left( {0,\tau_{res}^{2} } \right)} \hfill \\ \end{array} \hfill \\ \hfill \\ \end{gathered}$$

We chose not to centre age in this example as we were interested in the effect of the SNP at age zero. The choice to adjust for a cubic term of age was made based on evidence suggesting that each additional minor allele (A) of this variant is inversely associated with BMI from ages 0 to 3 and positively associated from ages 5.5 to 13 [5]. Effect sizes were estimated at multiple time points within the same cohorts, so we used generalized weights to adjust standard errors for the sample overlap [20].

## Results

### Scenarios 1 and 3: data generated with no age-varying genetic effect

#### Estimation of main genetic effect (${\beta }_{SNP}$) (i.e., the effect in a population with mean age = *10)*

**Fig. 2:**
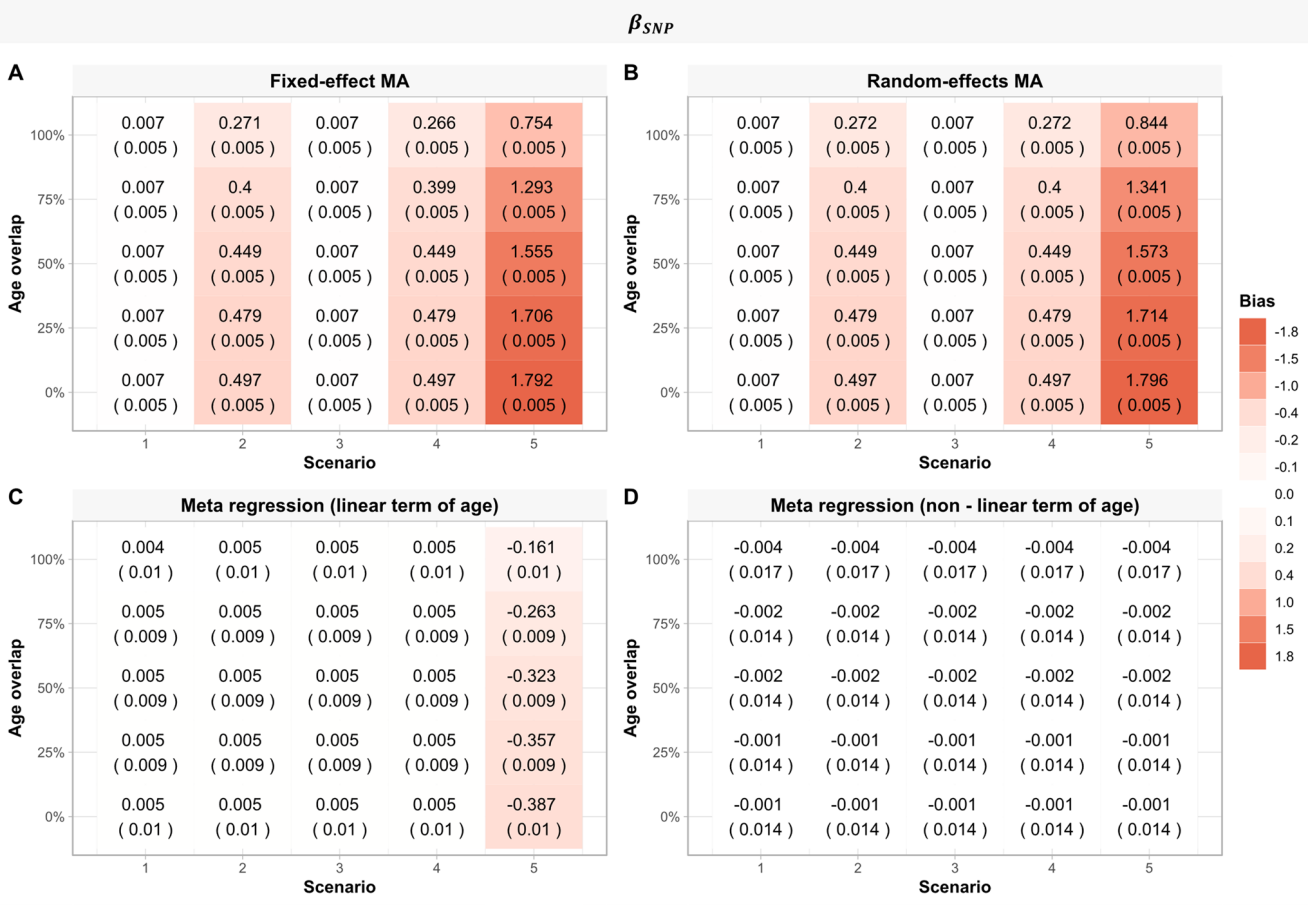
Heat maps displaying the mean difference (Monte Carlo standard error) between estimated main genetic and simulated main genetic effect (i.e., the genetic effect in a population of mean age = 10) (${\beta }_{SNP}$) for each method, scenario, and age overlap ( N = 1000). MA: Meta-analysis

As expected, as there was no age-varying genetic effect, both the fixed-effect and the random effects meta-analyses yielded estimates of the main genetic effect (${\upbeta }_{\mathrm{SNP}}$) similar to the simulated values across all proportions of overlapping ages between simulated studies (Fig. 2a, b), although CI coverage was below the nominal 95% level for the fixed-effect meta-analysis. Similarly, meta-regression models including either a linear or a quadratic term of age demonstrated negligible bias for the main genetic effect (Fig. 2c, d). CI coverage was consistent with the nominal 95% level for random-effects meta-analysis and both meta-regression models (Online Resource 1, Table S3).

#### Estimation of age-varying genetic effects (${\beta }_{SNP\times Age}, {\beta }_{SNP\times {Age}^{2}}$)

**Fig. 3:**
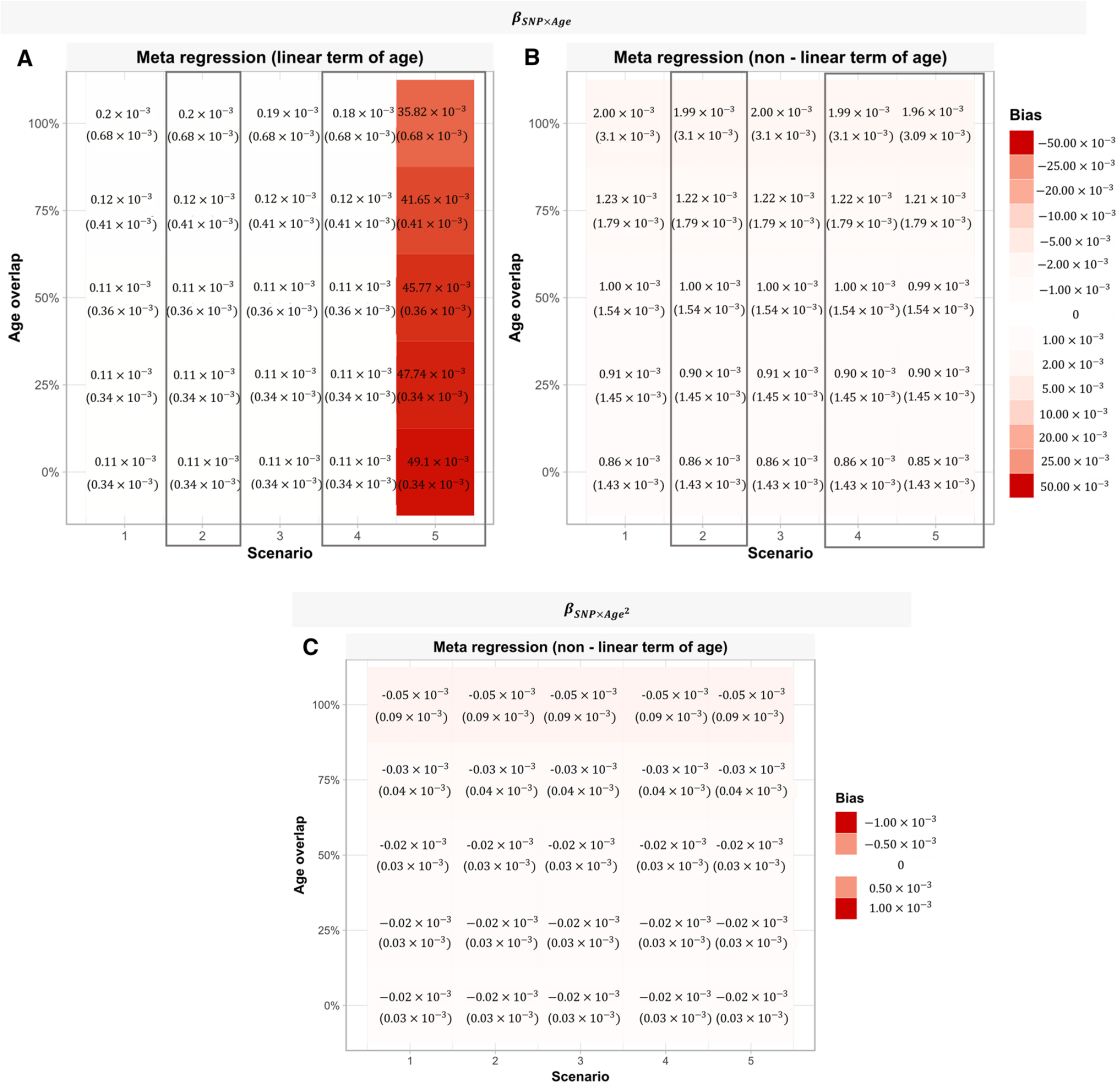
**A**, **B** Heat maps displaying the absolute bias (Monte Carlo standard error) of each meta-regression model, for each scenario and age overlap for the linear age-varying genetic effect (${\beta }_{SNP\times Age}$)(N = 1000). Squares represent scenarios where ${\beta }_{SNP\times Age}=0.02$ (scenario 2&3) and ${\beta }_{SNP\times Age}=0.04$ (scenario 5). In other scenarios${\beta }_{SNP\times Age}=0$. **C** Heat maps displaying the absolute bias (Monte Carlo standard error) of meta-regression including a non-linear term of age, for each scenario and age overlap for the non-linear age-varying genetic effect (${\beta }_{{SNP\times age}^{2}}$) (N = 1000). In scenario 5${\beta }_{{SNP\times age}^{2}}=0.001$. In all other scenarios${\beta }_{{SNP\times age}^{2}}=0$

Both meta-regression models (including a linear or quadratic term of age) yielded unbiased (i.e., mean of zero) estimates of the linear age-varying genetic effect (${\upbeta }_{\mathrm{SNP}\times \mathrm{Age}}$) (Fig. 3a, b) and the non-linear age-varying genetic effect (${\upbeta }_{\mathrm{SNP }\times {\mathrm{Age}}^{2}}$) (Fig. 3c). However, for both estimands (${\upbeta }_{\mathrm{SNP }\times \mathrm{ Age}}$,${\upbeta }_{\mathrm{SNP }\times {\mathrm{Age}}^{2}}$), the values estimated by the meta-regression models (erroneously including a linear or a quadratic term of age) were variable as seen by the large Monte Carlo SEs of bias in (Fig. 3a–c). As the proportion of overlapping ages between simulated studies was increased, the variability of estimated values increased.

CI coverage for the linear age-varying genetic effect (${\upbeta }_{\mathrm{SNP }\times \mathrm{ Age}}=0$) was consistent with the nominal 95% level for meta-regression models including a linear term of age, CI coverage was below the nominal 95% level for the linear and non-linear age-varying genetic effect (${{\upbeta }_{\mathrm{SNP }\times \mathrm{ Age}},\upbeta }_{\mathrm{SNP }\times {\mathrm{Age}}^{2}}$) in meta-regression models including a quadratic term of age (Online Resource 1, Table S4 and S5).

### Scenarios 2 and 4: data generated with a linear age-varying genetic effect

#### Estimation of main genetic effect (${\beta }_{SNP}$) (i.e., the effect in a population with mean age = 10)

When there were linear age-varying genetic effects, the difference between the estimated main genetic effects and the simulated value was not zero for both fixed and random-effects meta-analyses, across all proportions of overlapping ages between simulated studies (Fig. 2a, b). Meta-regression models including a linear or quadratic term of age produced unbiased estimates of the main genetic effect (Fig. 2c, d). As the age overlap between studies increased, the meta-regression estimates were more variable, as seen by the large Monte Carlo SEs.

In both scenarios, CI coverage for the main genetic effect (i.e., the effect if the population had a mean age of 10) was consistently below the nominal 95% level for fixed-effect and random-effects meta-analyses, across all proportions of overlapping ages between simulated studies. Both meta-regression models (including a linear or a non-linear term of age) yielded coverage of CIs consistent with the nominal 95% level (Online Resource 1, Table S3).

#### Estimation of age-varying genetic effect (${\beta }_{SNP\times Age}, {\beta }_{SNP\times {Age}^{2}}$)

Meta-regression estimates of the linear age-varying genetic effect (${\upbeta }_{\mathrm{SNP}\times \mathrm{Age}}$) were unbiased in both meta-regression models (Fig. 3a, b). Similarly, estimates of the non-linear age-varying genetic effect (${\upbeta }_{\mathrm{SNP }\times {\mathrm{Age}}^{2}}=0$) was unbiased in the meta-regression model including a quadratic term of age (Fig. 3c). The variance of the estimated values increased as the proportion of overlapping ages between simulated studies increased.

In both scenarios, CI coverage for the linear and non-linear age-varying genetic effects (${\upbeta }_{\mathrm{SNP }\times \mathrm{ Age}}$, ${\upbeta }_{\mathrm{SNP }\times {\mathrm{Age}}^{2}}$) were consistent with the nominal 95% level for meta-regression models including a linear term of age but not for meta-regression including a quadratic term of age (Online Resource 1, Table S4 and S5).

### Scenario 5: data generated with a quadratic age-varying genetic effect

#### Estimation of main genetic effect (${\beta }_{SNP}$) (i.e., the effect in a population with mean age = 10)

The difference between the estimated main genetic effect and the simulated value was not zero for both fixed and random meta-analyses (Fig. 2a, b). Meta-regression with only a linear age term gave biased estimates of the main genetic effect (Fig. 2c), due to wrongly specifying a linear meta-regression model. Meta-regression including a quadratic term of age yielded unbiased estimates, but variability of bias increased as age overlaps between studies increased (Fig. 2d).

CI coverage for the main genetic effect (i.e., the effect in a population of mean age = 10) was consistently below the nominal 95% level for all compared methods, except meta-regression models including a quadratic term of age, across all proportions of overlapping ages between simulated studies. (Online Resource 1, Table S3).

#### Estimation of age-varying genetic effect (${\beta }_{SNP\times Age}, {\beta }_{SNP\times {Age}^{2}}$)

Meta-regression models including only a linear term of age gave biased estimates of the linear age-varying genetic effect (${\upbeta }_{\mathrm{SNP }\times \mathrm{ Age}}$), across all proportions of overlapping ages between simulated studies, due to wrongly specifying a linear meta-regression model. In contrast, the meta-regression model also including a quadratic term of age yielded unbiased estimates of both the linear and non-linear age-varying genetic effects (${\beta }_{SNP \times Age}$), (${\upbeta }_{\mathrm{SNP }\times {\mathrm{Age}}^{2}}$), but variance of estimates increased as proportions of overlapping ages between studies increased (Fig. 3a–c).

CI coverage for the linear and non-linear age-varying genetic effect (${\upbeta }_{\mathrm{SNP }\times \mathrm{ Age}}$, ${\upbeta }_{\mathrm{SNP }\times {\mathrm{Age}}^{2}}$) was consistently slightly below the nominal 95% level in both meta-regression models (Online Resource 1, Table S4 and S5).

### Comparison of empirical SE and mean SE

Across all scenarios and proportions of overlapping ages between studies and for all estimands of interest, the random-effects meta-analysis and meta-regression including both a linear term and quadratic term of age yielded comparable empirical and mean SEs (Online Resource 1, Table S3-S5). In contrast, the fixed-effect meta-analysis produced mean SEs that were smaller than the empirical SEs, highlighting the incompatibility of fixed-effect meta-analysis to our data-generating mechanisms.

**Fig. 4:**
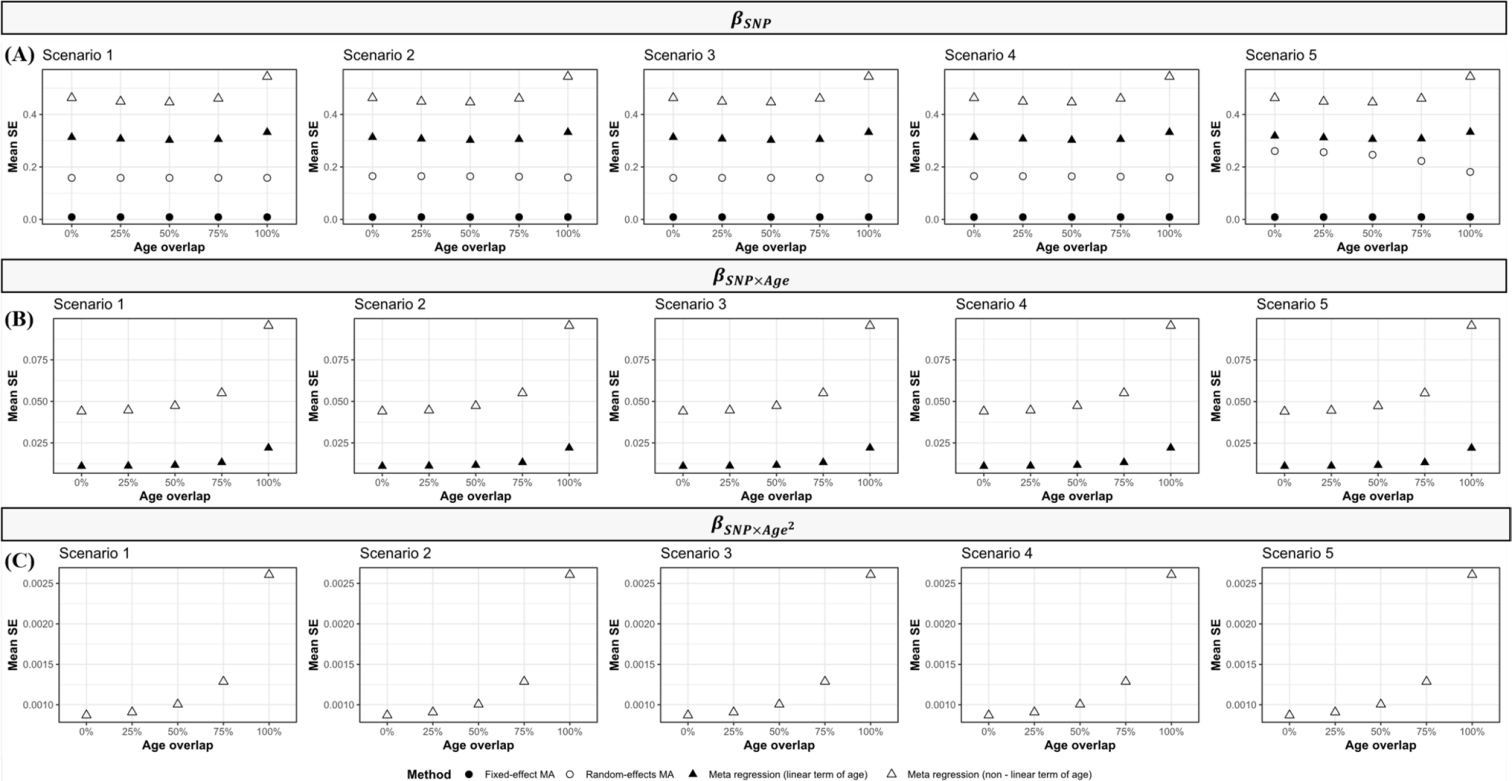
Mean Standard Errors for **A** the main genetic effect (${\beta }_{SNP}$), **B** linear and **C** non-linear age-varying genetic effects (${{\beta }_{SNP\times Age}, \beta }_{{SNP\times age}^{2}}$), for each method, scenario, and age overlap (N = 1,000)

The random-effects meta-analysis and meta-regression (both including a linear and quadratic term of age) produced large mean SEs of the main genetic effect (${\beta }_{SNP}$). However, when there was no age-varying genetic effect, meta-regression produced larger mean SEs compared to random-effects meta-analysis (Fig. 4a, Scenario 1 and 3). Including highly age-diverse studies (no (0%) age overlaps in age ranges between studies) in the meta-regression models produced more precise estimates of the main genetic effect compared to inclusion of less age-diverse studies (100% overlap in age ranges) (Fig. 4).

The meta-regression models including a linear term of age produced smaller mean SEs for both the linear and non-linear age-varying effects (${\upbeta }_{\mathrm{SNP }\times \mathrm{ Age}}$, ${\upbeta }_{\mathrm{SNP }\times {\mathrm{Age}}^{2}}$) compared with meta-regression models including a quadratic term of age, as expected due to having fewer number of parameters estimated (Fig. 4). Moreover, including highly age-diverse studies (no (0%) overlap in age ranges between studies) in the meta-regression models produced more precise estimates of the linear and non-linear age-varying effects compared to inclusion of less age-diverse (100% overlap in age ranges) studies.

### Influence of study characteristics

The Online Resource 1 (Table S6–S33) shows results from simulations varying the number of studies included in the analysis (from 10 to 80), sample sizes of each cohort (from 1,000 to 10,000), study level error (${u}_{j}$), individual level error (${\varepsilon }_{ij}$), standard deviations of age and centering age to weighted mean age across all studies. A small number of studies included in the meta-regression models (including a linear or quadratic term of age) resulted in CI coverage below the nominal 95% level for all estimands of interest (${\beta }_{SNP}$,${\upbeta }_{\mathrm{SNP }\times \mathrm{ Age}}$, ${\upbeta }_{\mathrm{SNP }\times {\mathrm{Age}}^{2}}$), even when the meta-regression models correctly reflected the data generating mechanisms. Increasing the number of participants within each cohort resulted in decreased mean SEs in fixed effect meta-analysis, while the results remained similar in random-effects methods. As expected, increasing study-level variability resulted in increased mean SEs in all random-effects methods, while mean SEs in fixed-effect meta-analysis remained unchanged. Conversely, increasing individual-level variability resulted in increased mean SEs in fixed effect meta-analysis and SEs remained unaffected in random-effects methods. When standard deviations of age were simulated to differ across studies all methods produced results similar to these observed in the “Base case scenario”. Lastly, we set the simulated value for the main genetic effect as the effect of genotype on phenotype at the weighted mean age across all studies. Meta-analysis and meta-regression models yield unbiased estimates of the main genetic effect $({\beta }_{SNP})$ in scenarios 1 to 4 (as expected), where there was either no age-varying genetic effect or a linear age-varying genetic effect in the data generating mechanism. Moreover, meta-analysis and meta-regression models including a linear term of centred age yield similar mean SEs. However, in scenario 5, where there was a non-linear age-varying genetic effect in the data generating mechanisms, meta-analysis and meta-regression including only a linear term of centred age produced biased estimates of the main genetic effect ${(\beta }_{SNP})$. Meta-regression results for the linear and non-linear age-varying genetic effects ${(\upbeta }_{\mathrm{SNP }\times \mathrm{ Age}}$, ${\upbeta }_{\mathrm{SNP }\times {\mathrm{Age}}^{2}})$ remained unchanged as expected.

### Estimating the age-varying genetic association between the rs9939609 SNP at the FTO locus and body mass index (BMI)

**Fig. 5:**
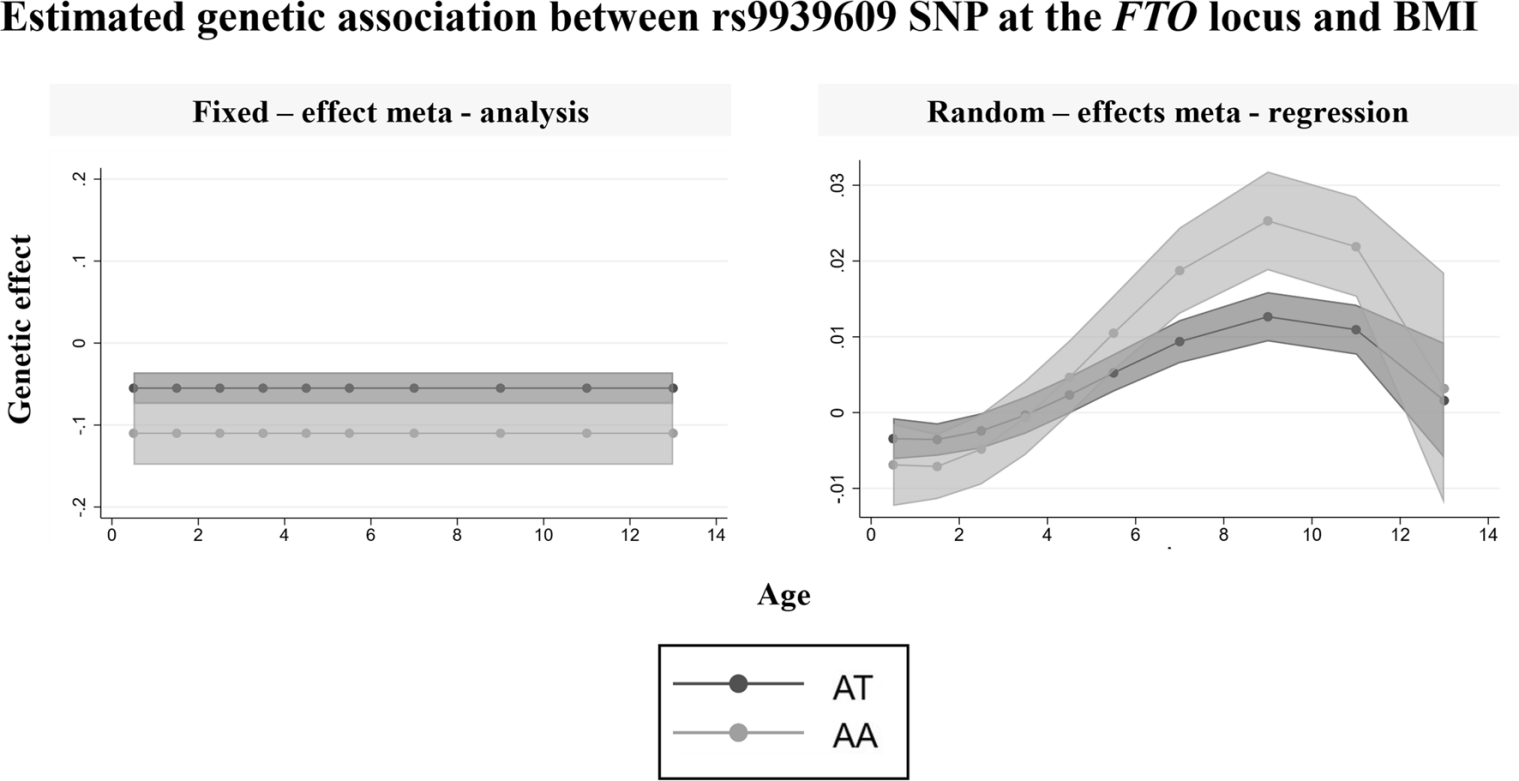
Estimated genetic association between rs9939609 SNP at the FTO locus and BMI, as estimated using fixed-effect meta-analysis and meta-regression adjusting for cubic term of age. Number of studies = 8, N = 19,725

Detailed information about the effect sizes used in the meta-analysis and meta-regression can be found in Online Resource 1 (Table S2). When we applied fixed-effect meta-analysis, a constant negative association (β = −0.05, 95% CI −0.06 to −0.03) between each additional minor allele (A) of rs9939609 and BMI was estimated. As fixed-effect meta-analysis is a weighted average of all studies, the estimated genetic effect is highly influenced by the fact that most of the largest studies are in early ages, therefore if a different selection of studies was used a different effect may have been estimated. In contrast, when we applied meta-regression, we observed an age-varying association: each additional minor allele (A) of rs9939609 was inversely associated with BMI at ages 0 to 3, and positively associated with BMI at ages 5.5 to 13 (Fig. 5). The percentage of variation between studies that can be attributed to heterogeneity rather than chance in fixed-effect meta-analysis was substantial (I^2^ = 76.9%), while adjusting for cubic age using meta-regression reduced the between study heterogeneity (I^2^ = 28.09%) (Online Resource 1, Table S34). Lastly, the association estimated using meta-regression was similar to the association described in the study we extracted summary data from [5]. In that study, individual participant data were utilised to model the median BMI curves of each genotype using the LMS method, and it was observed that carriers of minor alleles (A) showed lower BMI in infancy and higher in childhood.

## Discussion

In this study, we compared the performance of meta-regression and meta-analysis in accurately estimating main genetic effects (at age 10) and age-varying genetic effects (i.e., SNP-age interactions) from simulated cross-sectional GWAS studies and provided an empirical demonstration of this. Our results demonstrated that fixed-effect and random-effects meta-analyses accurately estimate genetic effects when these are not moderated by age, but not when age-varying genetic effects exist. This is because when there is age-moderation of genetic effects, the fixed or random-effects meta-analyses estimate the average effect across the (weighted) age distribution of the studies included. This will coincide with the genetic effect at the mean age if the genetic effect varies linearly with age, but not otherwise. In contrast, meta-regression produces unbiased estimates of both the main genetic effects and the age-varying genetic effects, regardless of whether age is a moderator or not. For example, in our empirical analysis, meta-analysis suggested an inverse association in children aged 0 to 13 years, whereas meta-regression correctly revealed an inverse association in early childhood (0 to 3 years) with this changing to a positive association between age 5.5 and 13 years. This empirical analysis demonstrates that the assumption that genetic effects are consistent across life will not always be the case, and we suggest that meta-regression should be used more widely to explore this possibility. It is important to note however that applying meta-regression when there are no age-varying genetic effects will produce less precise estimates, as more parameters will be estimated.

Exploring age-varying genetic effects in GWAS is important for various reasons. Firstly, it could help to better characterise the extent to which genetic variants which have already been associated with traits in a cross-sectional GWAS also influence change in that phenotype over time. For example, the FTO gene has been consistently reported to be associated with BMI and adiposity related traits, and there is evidence to suggest that this association may be time dependent [18, 19]. More specifically, a longitudinal cohort study reports association of the FTO gene with BMI during childhood and up to 20 years of age, when this association starts to get weaker with increasing age [4]. Secondly, it could contribute to identifying novel genetic variants, which may be associated with traits only in specific time periods during the life course. For instance, the LEPR locus has been associated with BMI in infancy and it is not linked with adult BMI, suggesting that its effect is no longer present in adulthood [21, 22]. Other traits with age-varying genetic effects are: diastolic blood pressure (DBP), systolic blood pressure (SBP), mean arterial pressure (MAP) and pulse pressure (PP), where genetic variants located in the *EHBP1L1* (DBP and MAP), *CASZ1* (SBP and MAP), and *GOSR2* (PP) loci had effects with opposite directions in the young versus old participants [8]; prostate-specific antigens (PSA) where 15 novel genetic variants were identified after accounting for age-varying genetic effects [6]; age-related macular degeneration where 4 loci, 2 of which were novel, identified after accounting for age-varying genetic effects [7]. Exploring age-varying genetic effects could therefore contribute to identifying novel genetic variants associated with age of onset, development of traits over time, and disease progression. Thirdly, the increasing number of GWAS and the public availability of their results has increased the popularity of two-sample MR studies, where the effect estimates of the genetic variants of exposure and outcome are extracted from different GWAS [11]. These allow the estimation of causal effects without requiring the exposure and outcome to be measured in the same participants. The causal effects estimated by MR are often interpreted as “lifetime causal effects of exposures on outcomes”. This interpretation has recently been challenged [23]. More specifically, when the association between genetic variants and exposure is time-varying, then the estimated causal effects that do not take account of this are likely to be biased, particularly if this is interpreted as a lifetime causal effect [23]. Therefore, exploring and accurately estimating age-varying genetic effects could help in better characterising causal relationships when the exposure of interest is time-varying.

Age-varying genetic effects could be explored in a GWAS in two different ways. The first approach would be to estimate SNP by age interactions within each GWAS. This would be possible if either the study was longitudinal, or it included participants with a wide range of ages. However, interaction analysis within GWAS might not always be feasible due to the large sample sizes required to have sufficient statistical power to identify interaction effects and the large computational time required to run the analyses. The study-specific SNP by age interactions would then be meta-analysed to obtain combined estimates [12]. This approach is feasible only in consortia where a pre-specified analysis plan requires estimation of interaction effects. Outside the scope of a consortium this approach would not be feasible, as GWAS rarely perform interaction analysis or report the full summary results of this analysis so they can be used in a meta-analysis. In this study we propose a second approach, meta-regression. Compared to meta-analysis of interaction effects, meta-regression will have smaller statistical power to identify interaction effects when median/mean ages across studies do not differ substantially [24]. Furthermore, as demonstrated in our simulations, meta-regression produces larger mean SEs compared to meta-analysis for the main genetic effect, when no age-varying genetic effect exist, therefore statistical power to identify main genetic effects is also smaller. Therefore, meta-regression still offers the opportunity of exploring age-varying genetic effects without the need of additional data collection, as it requires only summary level data for the effect of each SNP on the outcome, the mean and standard deviation of age of each study. Given the large number of already published GWAS that include large sample sizes and the public availability of their summary data, meta-regression could be considered as a step towards investigation of age-varying genetic effects without the need for additional data collection.

Even though GWAS commonly include age-diverse samples, meta-regression is rarely used to explore the differences in SNP-phenotype associations due to age. We have identified only two studies that applied meta-regression to account for heterogeneity introduced due to age. A GWAS of bone mineral density (N = 30 studies with a total 66,628 participants) applied meta-regression by stratifying the participants in each study into subgroups based on age and adjusting for the median age of each subgroup [15]. Two loci (in *ESR1* and *RANKL*) demonstrated age-varying genetic effects, with stronger associations in older age groups**.** A GWAS of blood pressure traits (N = 9 studies and 55,796 participants) applied meta-regression and adjusted for median age of each contributing study; it identified 9 genetic variants with age-varying effects. SNPs located in *CASZ1*, *EHBP1L1*, and *GOSR2*, demonstrated the largest age-dependent effects, with the effect alleles increasing blood pressure traits in the younger ages and decreasing them in the older [8].

It is important to note that the use of meta-regression is not limited to exploration of age-varying genetic effects. Meta-regression is beginning to be used to explore sources of heterogeneity in large scale genetic studies, such as to account for variation in phenotype assessment [25].GWAS that include samples from diverse ancestry backgrounds are becoming more prevalent and meta-regression has also been used as an approach to explore differences in SNP-phenotype association due to ethnicity/ancestry [25–27]. Therefore, meta-regression could be useful in estimating interactions between genetic variants and various risk factors such as ancestry and sex [25–28].

**Fig. 6:**
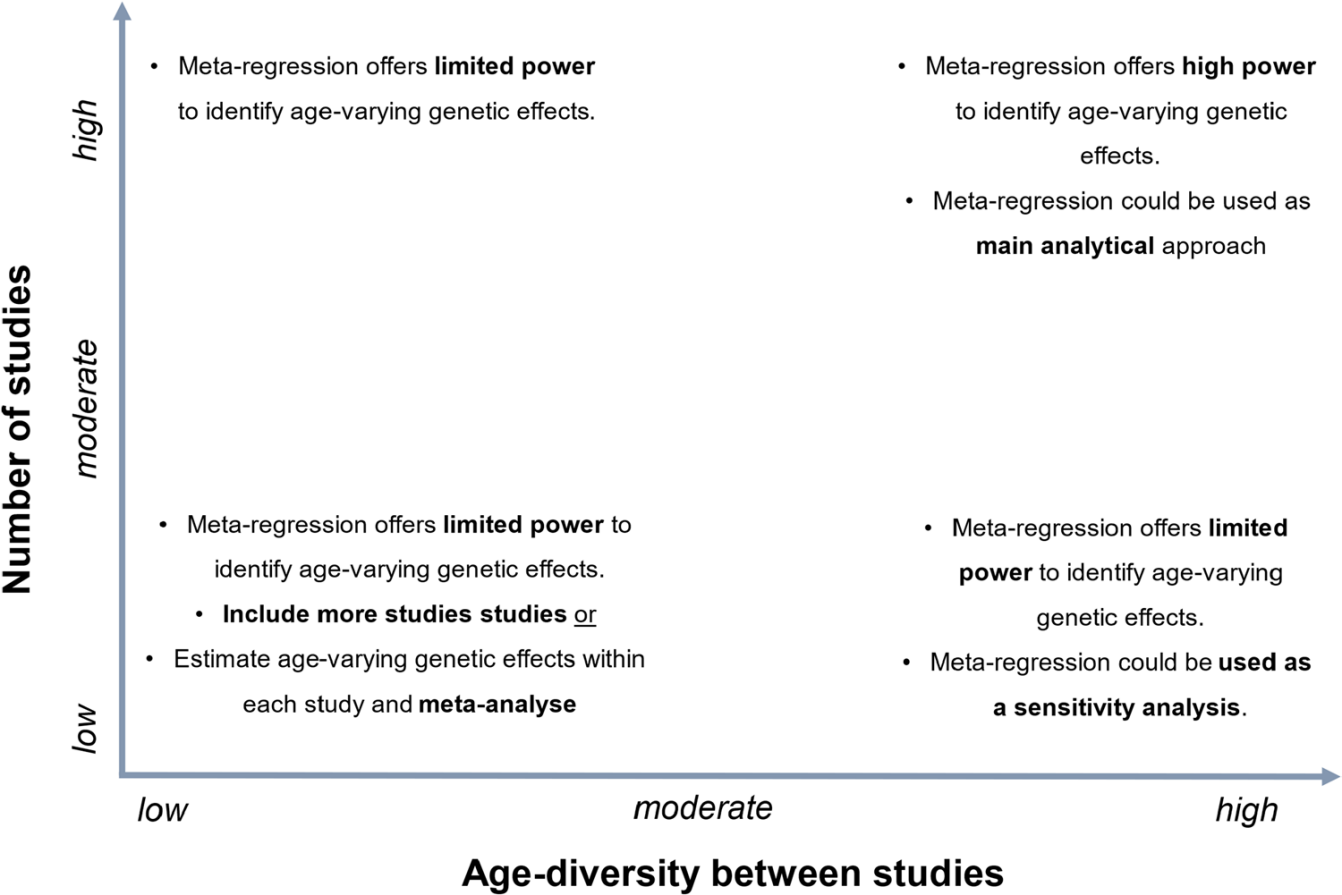
Recommendations for the application of meta-regression in estimating age-varying genetic effects in GWAS, based on the number of studies included and the age-diversity between studies

Meta-regression offers a feasible analytic tool to estimate age-varying genetic effects in the framework of GWAS. Similar to many statistical methods, a clear research question and justification for applying meta-regression is necessary a priori. We suggest two uses of meta-regression in a GWAS; (i) to complement GWAS meta-analysis to explore age-varying genetic effects and/or to (ii) estimate age-varying genetic effects when these are hypothesised and part of the main aim of the GWAS. We suggest that application of meta-regression should be considered in cases where moderate or high between-study heterogeneity (${I}^{2}\ge 25$) is observed. Additionally, careful consideration must be given regarding the data needed. Meta-regression requires only summary level data for the effect of each SNP on the outcome within each study, in addition to the mean and standard deviation of age of participants in each study. As many GWAS on various traits and diseases have already been published and their summary level data are often publicly available, meta-regression maximizes the value of already existing studies to explore age-varying genetic effects. However, often GWAS consortia provide summary level data of each SNP across studies, but not separately by study. For example, in our applied example we originally planned to use publicly available summary data from GWAS consortia but were unable to find any that provided summary data by study. Future GWAS should therefore aim to publish study specific summary results (including the mean and standard deviation of age) to enable meta-regression. Our simulation study suggests that consideration should be given to the number of studies and the age-diversity between the studies included in the meta-regression. This is in line with simulation studies reporting that the statistical power to identify moderator effects in meta-regression depends on the number of trials, the spread of study means, sample sizes and residual heterogeneity [24, 29]. In Fig. 6, we provide guidance regarding the number of trials and age-diversity. When the number of studies included in the meta-regression is low ($\le 60$ in our simulations) and the age-diversity between samples is low (study mean ages are similar), meta-regression has limited power to estimate age-varying genetic effects. Therefore, researchers will need to either include more studies of age-diverse samples or estimate age-varying genetic effects within studies and meta-analyse these. The additional number of studies that needs to be included to reach adequate statistical power to identify main and age-varying genetic effects, can be calculated by performing sample size calculations. However, when the number of studies included in the meta-regression is high and the between-study age-diversity moderate to high, then meta-regression should be considered as the main analytical approach in GWAS. Lastly, our simulations suggest the importance of carefully selecting whether the application of a linear or non-linear meta-regression is appropriate, as over-misspecification of the model could lead to below nominal CI coverage and under-specification could lead to biased estimates. Information about the effect of a SNP on a phenotype, in relation to age, can be obtained by smaller longitudinal studies, where this relationship can be investigated.

Our study has limitations that should be considered. Even though we explored a wide range of plausible scenarios in our simulations, we have inevitably not explored all possible real-world scenarios. For example, further work would be needed to investigate the applicability of our results in cases where the trait of interest is binary/categorical or in cases where the sample size of studies differs and this differentiation is age related (e.g., smaller sample sizes in studies with older participants compared to studies with younger participants). Simulating larger number of studies and sample sizes would have been computationally intensive and would not provide any further insights, as we were already able to observe that power of meta-regression is influenced by the number of studies included. Additionally, we have only investigated the applicability of meta-regression in estimating the association between the genetic effects and quadratic function of age (non-linear age-varying genetic effects). Meta-regression could be easily extended to accommodate higher degree polynomials and splines. Moreover, we have not explored the impact of overlapping samples on our results. Overlapping participants between GWAS results in higher false positive rates and similarly will have an impact on meta-regression. Methods have been proposed to adjust for sample overlaps in a meta-analytic framework. For instance, in our empirical example each GWAS contributed summary level data in multiple time points, thus sample overlaps could not be avoided and to account for this overlap we used generalized weights to adjust standard errors [20]. Lastly, the impact of population stratification on our results has not been explored. Inadequate adjustment for population stratification will result in biased estimates of GWAS, which in turn will produce biased estimates in both meta-analysis and meta-regression. However, as it is common practice to adjust for population stratification in each study included in GWAS, we consider bias due to population stratification to be minimal.

## Conclusions

A correctly specified meta-regression analysis can provide unbiased estimates of the main and age-varying genetic effects using summary level data, particularly when summary level data are available for a large number of studies covering a range of ages.

### Supplementary Information

Below is the link to the electronic supplementary material.

Supplementary file1 (XLSX 345 kb)

Supplementary file2 (DOCX 26 kb)

## Data Availability

The code used in this simulation study is available at *https://github.com/Pagoni-P/GWAS-metareg.git*. Summary data used in the applied example can be found in the original publication (https://doi.org/10.1371/journal.pgen.1001307).
