# Supplementary material for: Meta-regression of genome-wide association studies to estimate age-varying genetic effects
Source: Eur J Epidemiol. 2024 Jan 6;39(3):257–70. doi: 10.1007/s10654-023-01086-1 (PMC10995067; doi:10.1007/s10654-023-01086-1)
Supplement: Supplementary file 2 — Supplementary file2 (DOCX 26 kb) [file 10654_2023_1086_MOESM2_ESM.docx]

**Meta-regression of Genome-Wide Association Studies to estimate age-varying genetic effects**

**Online resource 2**

Panagiota Pagoni^1,2^, Julian P. T. Higgins^1,2,3^, Deborah A. Lawlor^1,2^, Evie Stergiakouli^1,2^, Nicole M. Warrington^1,4,5,6^, Tim T. Morris†^7^, Kate Tilling†^1,2^

1 MRC Integrative Epidemiology Unit at the University of Bristol, Bristol, UK

2 Population Health Sciences, Bristol Medical School, University of Bristol, Bristol, UK

3 National Institute for Health Research Bristol Biomedical Research Centre, University of Bristol, Bristol, UK

4 Institute for Molecular Bioscience, University of Queensland, Brisbane, QLD Australia

5 Frazer Institute, University of Queensland, Brisbane, QLD Australia

6 K.G. Jebsen Center for Genetic Epidemiology, Department of Public Health and Nursing, NTNU, Norwegian University of Science and Technology, Trondheim, Norway

7 Centre for Longitudinal Studies, Social Research Institute, University College London, London, United Kingdom

† Joint last authors

**Correspondence to** Panagiota Pagoni, MRC Integrative Epidemiology Unit at the University of Bristol, Oakfield House, Oakfield Grove, Bristol, United Kingdom, BS8 2BN, email: [panagiota.pagoni@bristol.ac.uk](mailto:panagiota.pagoni@bristol.ac.uk)

**Note S1.** Estimation of combined genetic effect in fixed-effect and random-effects meta-analysis.

Both fixed-effect and random-effects meta-analyses provide estimates of the combined genetic effect of a given SNP across all studies ($\hat{\beta}_{SNP}$) and the between study variability ($\hat{\tau}^{2}$). The combined genetic effect is a weighted mean of the genetic effects across all studies:

$$\hat{\beta}_{SNP}=\frac{\sum_{j=1}^{n} W_{j}\times\hat{\beta}_{1j}}{\sum_{j=1}^{n} W_{j}}$$

where $W_{j}$ is the weight assigned to each study and $\hat{\beta}_{1j}$ is the estimated effect for given SNP in each study. The fixed-effect model has only one source of variance (within-study variance due to sampling error), while the random-effects model has two sources of variance (within- and between-study variance). Therefore, the study weights are $W_{j}=\frac{1}{{\hat{SE(\beta_{1j})}}^{2}}$ and $W_{j}=\frac{1}{{\hat{SE(\beta_{1j})}}^{2}+ \tau^{2}}$ for fixed-effect and random-effects models, respectively.

**Note S2.** Derivation of meta-regression models including quadratic and cubic terms of age.

**Meta-regression including a quadratic term of age**

Suppose we have $j=1,2,..,k$ studies with $i=1,2,..,n$ participants each. Then we define $Y_{ij}$ as:

| $Y_{ij}=\beta_{0}+\beta_{age}\times{age}_{ij}+\beta_{{age}^{2}}\times{age}_{ij}^{2}+{\beta_{SNP}\times SNP}_{ij}+{{\beta_{SNP\times age}\times{age}_{ij}\times SNP}_{ij}+\beta_{{SNP\times age}^{2}}\times{age}_{ij}^{2}{\times SNP}_{ij}+ \varepsilon}_{ij}$ | *(1)* |
| --- | --- |

Then within each study the estimated average genetic effect in each study can be expressed as:

| $\hat{\beta}_{1j}=\hat{\beta}_{SNP}\times SNP+\hat{\beta}_{SNP\times age}\times SNP\times\bar{{age}_{j}}+\hat{\beta}_{SNP\times{age}^{2}}\times SNP\times\bar{{age}_{j}^{2}}$ | *(2)* |
| --- | --- |

where $\beta_{SNP}$ is the mean genetic effect across studies, $\beta_{SNP\times age}$ is the difference in the mean effect of a given SNP for each one year increase in age, $\beta_{SNP\times{age}^{2}}$ is the difference in the mean effect of a given SNP for each one year difference in the square of age, mean of age $\bar{{age}_{j}}=E\left( {age}_{j} \right)$ and mean of quadratic age $\bar{{age}_{j}^{2}}=E({age}_{j}^{2})$.

Given that mean age ($\bar{{age}_{j}}$) and the variance of age ($Var\left( {age}_{j} \right)={SD({age}_{j})}^{2})$ are known for each cross-sectional GWAS, we can calculate the mean of quadratic age of each study ( $\bar{{age}_{j}^{2}})$ as follows:

$$Var\left( {age}_{j} \right)=E\left( {age}_{j}^{2} \right)-\left[ E({age}_{j}) \right]^{2}$$

$$\Longrightarrow E\left( {age}_{j}^{2} \right)= Var\left( {age}_{j} \right)+\left[ E({age}_{j}) \right]^{2}$$

$$\Longrightarrow\bar{{age}_{j}^{2}}= Var\left( {age}_{j} \right)+{\bar{{age}_{j}}}^{2}$$

**Meta-regression including a cubic term of age**

Suppose we have $j=1,2,..,k$ studies with $i=1,2,..,n$ participants each. Suppose we have $j=1,2,..,k$ studies with $i=1,2,..,n$ participants each. Then we define $Y_{ij}$ as:

| $Y_{ij}=\beta_{0}+\beta_{age}\times{age}_{ij}+\beta_{{age}^{2}}\times{age}_{ij}^{2}+\beta_{{age}^{3}}\times{age}_{ij}^{3}+{\beta_{SNP}\times SNP}_{ij}+{{\beta_{SNP\times age}\times{age}_{ij}\times SNP}_{ij}+\beta_{{SNP\times age}^{2}}\times{age}_{ij}^{2}{\times SNP}_{ij}+\beta_{{SNP\times age}^{3}}\times{age}_{ij}^{3}{\times SNP}_{ij}+ \varepsilon}_{ij}$ | *(3)* |
| --- | --- |

Then within each study the estimated average genetic effect in each study study can be expressed as:

| $\hat{\beta}_{1j}=\hat{\beta}_{SNP}\times SNP+\hat{\beta}_{SNP\times age}\times SNP\times\bar{{age}_{j}}+\hat{\beta}_{SNP\times{age}^{2}}\times SNP\times\bar{{age}_{j}^{2}}+\hat{\beta}_{SNP\times{age}^{3}}\times SNP\times\bar{{age}_{j}^{3}}$ | *(4)* |
| --- | --- |

where $\beta_{SNP}$ is the mean genetic effect across studies, $\beta_{SNP\times age}$ is the difference in the mean effect of a given SNP for each one year increase in age, $\beta_{SNP\times{age}^{2}}$ is the difference in the mean effect of a given SNP for each one year difference in the square of age, $\beta_{SNP\times{age}^{3}}$ is the difference in the mean effect of a given SNP for each one year difference in the cubic of age , mean of age $\bar{{age}_{j}}=E\left( {age}_{j} \right)$, mean of quadratic age $\bar{{age}_{j}^{2}}=E({age}_{j}^{2})$ and mean of cubic age $\bar{{age}_{j}^{3}}=E({age}_{j}^{3})$

Given that mean age ($\bar{{age}_{j}}$) and the variance of age ($Var\left( {age}_{j} \right)={SD({age}_{j})}^{2})$ are known for each cross-sectional GWAS, we can calculate the mean of cubic age of each study ( $\bar{{age}_{j}^{3}})$ assuming that age distribution is symmetrical within each study.

Skewness for a random variable X can be expressed as :

$$Skew= E\left[ \left( \frac{X-\mu}{\sigma} \right)^{3} \right]= 0$$

$$\Rightarrow\frac{E\left( X^{3} \right)-3\mu E\left( X^{2} \right)+3\mu^{2}E\left( X \right)-\mu^{3}}{\sigma^{3}}=0$$

$$\Rightarrow E\left( X^{3} \right)-3\mu\left[ E\left( X^{2} \right)+3\mu E\left( X \right) \right]-\mu^{3}=0$$

$$\Rightarrow E\left( X^{3} \right)-3\mu\sigma^{2}-\mu^{3}=0$$

$\Rightarrow E\left( X^{3} \right)=3\mu\sigma^{2}+\mu^{3}$

Therefore, if $X={age}_{j}$ then cubic age of each study can be expressed as:

| $\bar{{age}_{j}^{3}}=3\times\bar{{age}_{j}}\times Var\left( {age}_{j} \right)+{(\bar{{age}_{j}})}^{3}$ | *(4)* |
| --- | --- |
